# Rapid eye movement (REM) sleep microarchitecture is altered in patients with wake-up ischemic stroke: A polysomnographic study

**DOI:** 10.1016/j.nbscr.2021.100069

**Published:** 2021-06-23

**Authors:** Jaidaa Mekky, Osama El-Kholy, Eman Hamdy, Akram Fawzy

**Affiliations:** Department of Neuropsychiatry, Faculty of Medicine, Alexandria University, Alexandria, Egypt

**Keywords:** Sleep, Cerebrovascular stroke, Polysomnography, Daytime stroke, Wakeup stroke

## Abstract

It is well established that certain alteration of sleep disorders occur in patients with wake-up stroke (WUS) such as sleep disordered breathing, periodic limb movements and sleep duration. However, the data are lacking about the microarchitecture of different sleep stages among those patients.

**Aim of work:**

To compare the polysomnographic microarchitecture of rapid eye movement (REM) sleep between WUS and daytime stroke (DTS).

**Methods:**

A cross-sectional polysomnographic study was conducted on 20 patients with WUS and 20 patients with DTS, with analysis of REM sleep microarchitecture in specific.

**Results:**

Patients with WUS had significantly shorter REM stage (11.76 ± 5.48% in WUS versus 16.59 ± 5.33% in DTS, P = 0.008), longer early morning REM was (25.70 ± 13.13 min in WUS versus 4.15 ± 4.69 min in DTS, P=<0.001), higher apnea-hypopnea index (AHI) during REM (6.29 ± 10.18 in WUS versus 1.10 ± 4.57 in DTS, P = 0.009), and lower mean Oxygen saturation during REM (92.70 ± 3.63 WUS versus 95.45 ± 1.35 DTS, P = 0.012). The OR of early morning REM duration was 1.8 (CI 1.099–3.130, p = 0.021) for WUS.

**Conclusion:**

The microarchitecture of REM sleep is disrupted in patients with wake-up stroke.

## Highlights

⁃ The polysomnographic parameters are significantly different between patients with daytime stroke (DTS) and wake-up stroke (WUS).
⁃ Patients with WUS have longer stage 2 and shorter REM stage.
⁃ The REM microarchitecture is disrupted among patients with WUS e.g. early morning REM duration in comparison to patients with daytime stroke (DTS).
⁃ The distruption was in the form of shorter REM percentage to total sleep time, higher sleep apnea hypopnea index (AHI) during REM sleep, lower oxygen saturation during REM sleep and longer early morning REM duration.
⁃ Periodic limb movement (PLM) index, and PLM-related arousals are higher among DTS.

## 1 Introduction

Cerebrovascular stroke and sleep are closely correlated (Mims and Kirsch, 2016). Several pathophysiological mechanisms have been proposed in literature that explain the occurrence of stroke at high rates with certain sleep disorders (Koo et al., 2018). Obstructive sleep apnea (OSA) is on the top of the list of sleep disorders increasing the risk for cerebrovascular stroke (Yaggi et al., 2005). Obstructive sleep apnea increases stroke risk via several direct and indirect mechanisms. Direct mechanisms include increased sympathetic activity, nocturnal hypertension, intermittent hypoxia, arousal responses, hemodynamic instability, oxidative stress and endothelial dysfunction (Saper and Fuller, 2017; Verrier et al., 2005; Terplan et al., 1993). Indirect mechanisms include increasing the risk for other stroke risk factors such as diabetes mellitus, hypertension, and cardiac arrhythmias (Javier Nieto et al., 2000; Peppard et al., 2000; Yamashita et al., 1997). Insomnia, periodic limb movements (PML), and restless leg syndrome (RLS) are other sleep disorders that increase stroke risk (Koo et al., 2018). They were proposed to increase the stroke risk via increasing sympathetic activity, micro-arousals, oxidative stress, hypoxia, inflammation and metabolic dysregulation (Koo et al., 2018). Sleep duration was also reported to be related to stroke. Both long and short sleep duration were reported to increase sleep risk (Chen et al., 2008; Helbig et al., 2015).

Though the relationship between stroke and sleep is well established, data is scarce about the sleep stages microarchitecture in patients with wake-up (WUS) and patients with daytime stroke (DTS). The sleep architecture was compared between patients with wake-up stroke (WUS) and patients with daytime stroke (DTS) in several literature studies, and the data from these studies are conflicting (Hsieh et al., 2012; Šiarnik et al., 2016; Mohammad et al., 2019; Xiao et al., 2018). Most of these studies focused on sleep disordered berating (SDB) and OSA, reporting them as risk factors for wake-up stroke. In this research, we propose a directional relationship between rapid eye movement (REM) sleep disturbances and the development of WUS. To test this hypothesis, we studied and compared the REM sleep microarchitecture between WUS and DTS patients among a sample of Egyptian population.

## 2 Method and materials

### 2.1 Study design and patients

This was a cross-sectional study conducted on 40 patients with cerebrovascular stroke who were admitted at the department of neurology at Alexandria university hospitals in Egypt during the period from June 2019 to June 2020. Twenty of these patients had their stroke onset on awakening, and the remaining 20 had their stroke onset during the daytime. Inclusion criteria included patients older than 45 years diagnosed with ischemic thrombotic cerebrovascular stroke within one week with a National Institutes of Health Stroke Scale (NIHSS) of 20 or less. Patients with stroke in young age, embolic stroke, or Glasgow coma scale less than 11 were excluded from the study. Patients with co-morbid psychiatric illness affecting sleep like depression or anxiety, patients on medications that affect sleep e.g., hypnotics or stimulants, and patients with pre-existing documented sleep disorders were excluded from this study.

### 2.2 Demographic and clinical data

All patients were subjected to complete history taking, neurological examination, laboratory profile, brain imaging, and NIHSS assessment. An Arabic validated version of Pittsburg sleep quality index (PSQI) was administered for all patients for self-reporting to evaluate the sleep quality. Demographic, clinical, laboratory, and radiological data were collected from all patients.

### 2.3 Polysomnography

An overnight polymnographic (PSG) study was performed to all patients within one week of stroke onset. The PSG study was conducted using the data acquisition system Cadwell (Version 2.1, USA) at the sleep laboratory of the department of neurology at Alexandria University Hospital. The PSG recordings included four channels of EEG, two lateral canthus eye leads for electrooculogram (EOG), two submental electromyography (EMG) leads, two electrocardiogram (ECG) leads, one nasal thermistor for airflow, one channel for oxygen saturation (SO_2_), chest and abdominal belts to monitor respiratory movements, and two anterior tibialis EMG leads. Two independent sleep specialists scored the PSG records according to the American Academy Standard Manual (AASM) for the scoring of sleep-associated events (2020) (Scoring Manual - Ame, 2020). The sleep parameters collected for analysis were total sleep time (TST), wakefulness after sleep onset (WASO), sleep efficiency, sleep latency, REM latency, percentages of different sleep stages (i.e. stage I, II, slow-wave sleep and rapid-eye movement (REM) sleep), early morning REM duration, arousal index, total apnea-hypopnea index (AHI), AHI during non-rapid eye movement (NREM) sleep, AHI during REM sleep, mean O_2_ during sleep, mean O_2_ during REM sleep, mean O_2_ during NREM sleep, lowest O_2_ during sleep, and the stage when the lowest O_2_ saturation occurred, snoring index, and periodic limb movement index (PLM) index.

### 2.4 Statistical analysis

All data were fed to the computer and analyzed using IBM SPSS software package version 20.0 (Armonk, NY: IBM Corp). Qualitative data were described using numbers and percent. The Kolmogorov-Smirnov test was used to verify the normality of distribution. Quantitative data were described using range (minimum and maximum), mean, standard deviation, median, and interquartile range (IQR). The significance of the obtained results was judged at the 5% level. The used tests were Chi-square test for categorical variables to compare between different groups, Fisher's Exact or Monte Carlo correction for chi-square when more than 20% of the cells have expected count less than 5, student t-test for normally quantitative variables to compare between two studied groups, and Mann Whitney test for abnormally quantitative variables to compare between two studied. The statistical power of the results was 88.7%, and it was calculated using G*power 3.1.9.4 program (Erdfelder et al., 2009).

### 2.5 Ethical consideration

Ethical approval was obtained from the Ethics Committee of the Alexandria Faculty of Medicine, which has FWA from 2010 and operates according to the ICH GCP guidelines and applicable local and institutional regulations and guidelines.

## 3 Result

### 3.1 Demographic and clinical data

Twenty patients with DTS (F:M = 85%:15%), and 20 patients with WUS (F:M = 60%:40%) were recruited to this study (P = 0.077). The mean age of the patients with DTS was 57.45 ± 11.25 years and the mean age of the patients with WUS was 61.10 ± 10.90 (P = 0.304). A comparative analysis of the sociodemographic, clinical, and radiological characteristics of the studied groups is detailed in Table 1. Both demographic and clinical variables were comparable between the two studied groups (p > 0.05).

**Table (1):** A comparative analysis of WUS and DTS sociodemographic, clinical, and radiological data (n = 40).

	WUS (n = 20)	DTS (n = 20)	P-value
Sociodemographic data			
Gender: Male (%)	60.0	85.0	0.077
Age (Mean ± SD)	61.10 ± 10.90	57.45 ± 11.25	0.304
BMI (Mean ± SD)	30.41 ± 1.45	29.51 ± 1.84	0.095
**Risk factors**			
Hypertension (%)	55.0	75.0	0.185
Diabetes (%)	25.0	40.0	0.311
Ischemic heart disease (%)	20.0	0.0	0.106
Smoking	55.0	45.0	0.527
Family history of stroke/TIA	0.0	5.0	1.000
**Clinical presentation**			
Facial weakness (%)	95.0	85.0	0.605
Dysarthria (%)	75.0	75.0	1.000
Sensory symptoms (%)	10.0	10.0	1.000
Aphasia (%)	0.0	5.0	1.000
Gaze palsy (%)	20.0	10.0	0.661
Ataxia (%)	5.0	0.0	1.000
Hemiparesis (%)	95.0	90.0	1.000
NIHSS (Mean ± SD)	7.60 ± 4.03	7.55 ± 4.20	0.968
**TOAST classification type of stroke**			
Lacunar stroke	50.0	55.0	0.752
Large vessel stroke	50.0	45.0	0.752
**Brain imaging findings**			
Parieto-temporal	30.0	15.0	0.451
Internal capsule	30.0	40.0	0.507
Thalamus	10.0	5.0	1.000
Basal ganglia	25.0	25.0	1.000
Brainstem	5.0	0.0	1.000

DTS: Daytime stroke, NIHSS: National Institutes of Health Stroke Scale, SBP: Systolic blood pressure, SD: Standard deviation, TOAST: Trial of Org 10172 in Acute Stroke Treatment, WUS: wake-up stroke.

### 3.2 Sleep quality and polysomnographic data

**Fig. 1:**
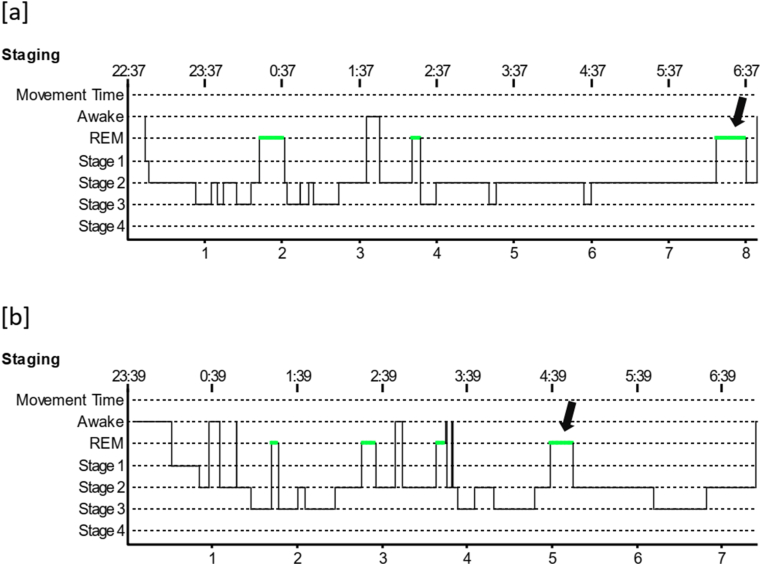
Hypnograms of a patient with WUS (a) and a patient with DTS (b) showing a longer early morning REM duration in WUS than DTS (arrows). Of note, Stage 2 sleep% is higher and REM sleep % is lower in WUS than DTS.

**Fig. 2:**
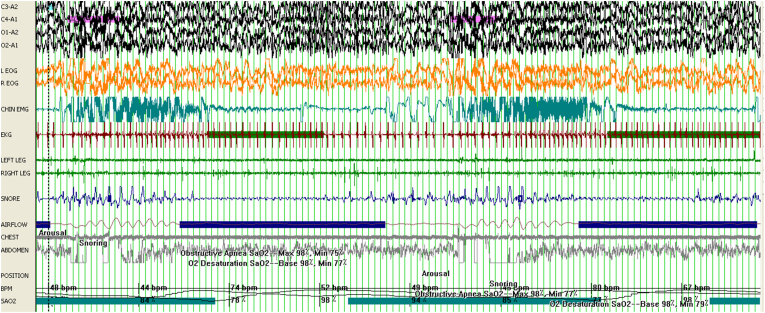
A polysomnographic epoch (120 s) of a patient with WUS showing several apneas during REM sleep and an oxygen saturation falling to 78%.

**Fig. 3:**
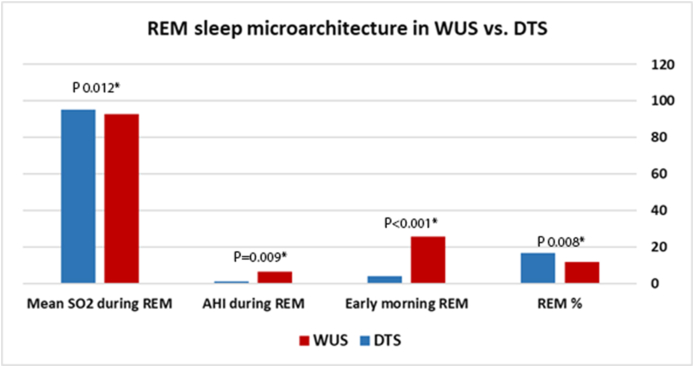
REM sleep microarchitecture in patients with WUS versus patients with DTS.

**Fig. 4:**
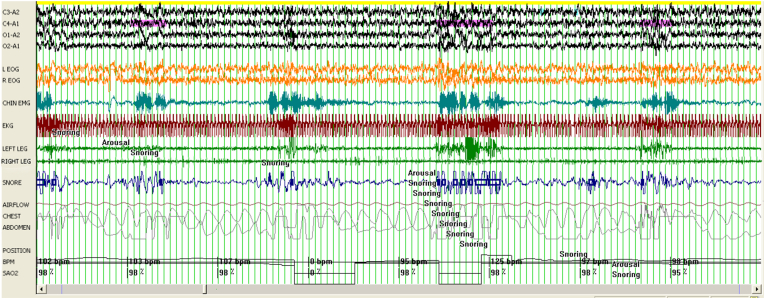
A polysomnographic epoch (120 s) of a patient with DTS showing a cluster of PLMs during stage 2.

There was no significant difference between PSQI scores among DTS and WUS patients (Table 2). With regards to polysomnographic data (Table 2), the differences encountered between DTS and WUS patients were stage 2 sleep %, REM sleep %, early morning REM duration (in minutes), AHI index during REM sleep, mean O_2_ saturation during REM, and PLM index. The mean percentage of stage 2 sleep was 60.09 ± 7.67 among patients with DTS and 66.67 ± 6.92 among patients with WUS (P = 0.018) (Fig. 1). The mean REM percentage was 16.59 ± 5.33 among DTS patients and 11.76 ± 5.48 among WUS patients (P = 0.008) (Fig. 1). The mean early morning REM duration was 4.15 ± 4.69 min among DTS patients and 25.70 ± 13.13 min among WUS patients (p=<0.001) (Fig. 1). The mean AHI during REM sleep was 1.10 ± 4.57 and 6.29 ± 10.18 among DTS and WUS patients, respectively (P = 0.009) (Fig. 2). The mean SO_2_ during REM sleep was 95.45 ± 1.35 and 92.70 ± 3.63 among DTS and WUS patients, respectively (P = 0.012) (Fig. 2). Fig. 3 summarizes the differences in REM sleep microarchitecture between patients with WUS and patients with DTS. The microarchitecture of NREM sleep was not significantly different between WUS and DTS (Table 2). The mean PLM index was 18.66 ± 24.46 and 7.45 ± 7.30 among DTS and WUs patients, respectively (P = 0.043). The mean PLM-related arousal index (Fig. 4) was 0.501 ± 0.52 and 0.18 ± 0.24 among DTS and WUS patients, respectively (P = 0.019). On regression analysis (Table 3), early morning REM duration (OR = 1.855, (CI 1.099–3.130)) had the highest OR for developing WUS.

**Table (2):**
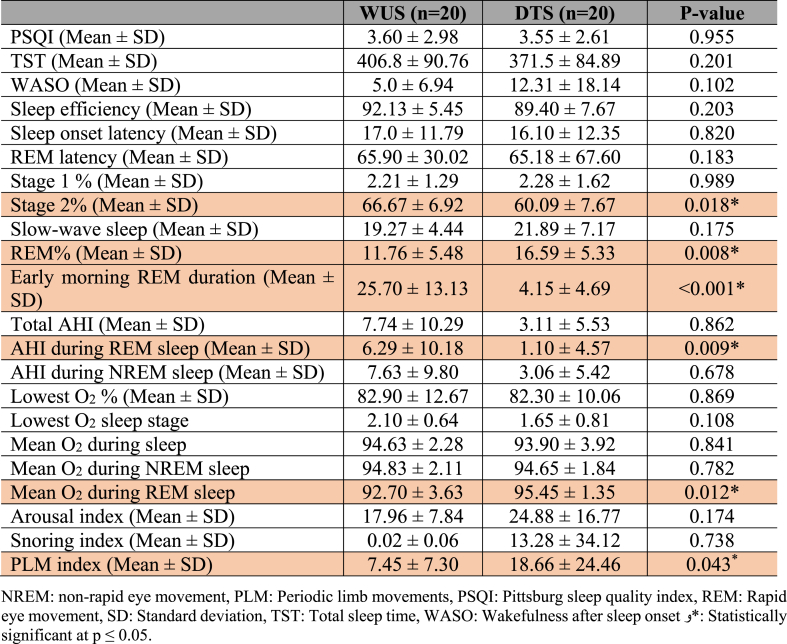
A comparative analysis of WUS and DTS PTSQI and polysomnographic variables.

**Table (3):**
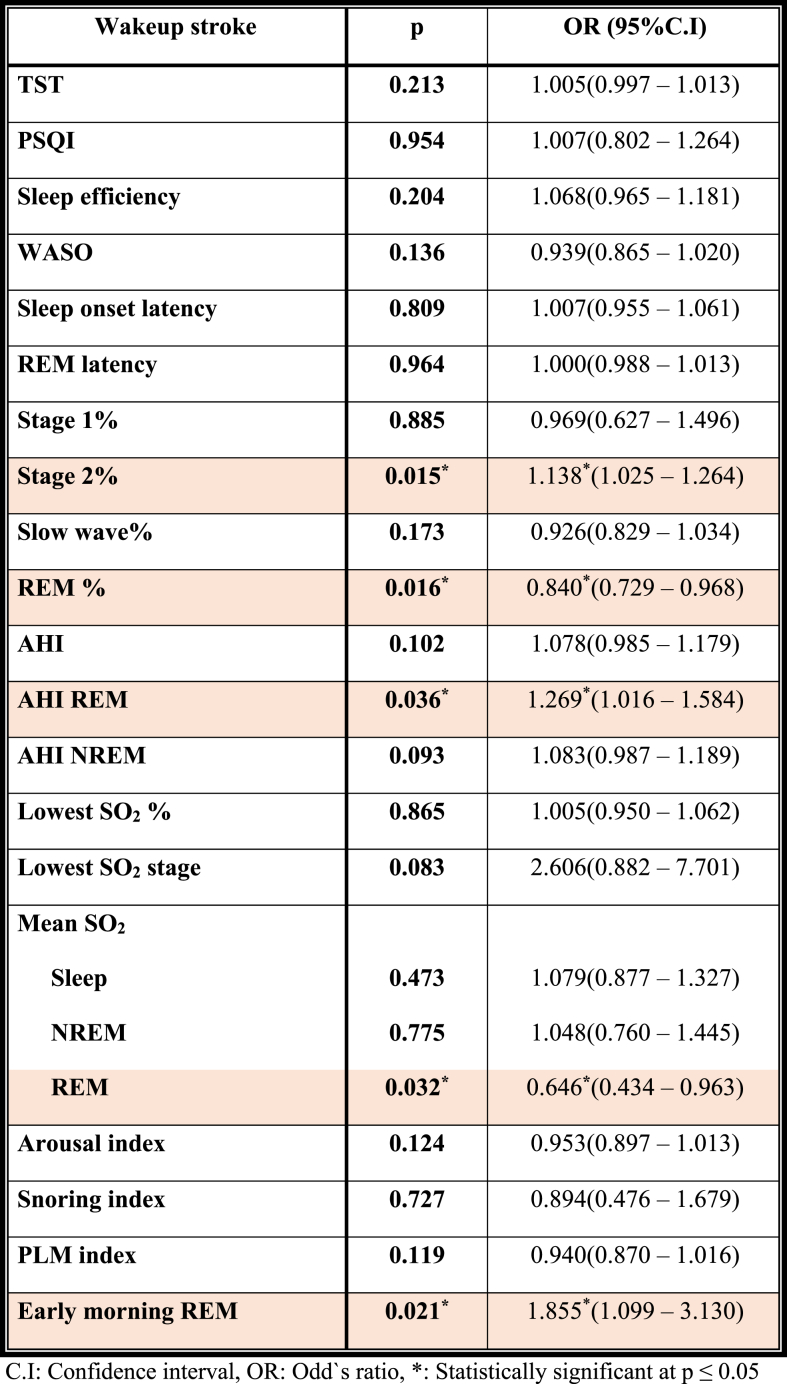
Regression analysis for the polysomnographic variables affecting wakeup stroke (n = 40) for total sample.

## 4 Discussion

It is well established that sleep and stroke are complexly related. Sleep is fragmented in patients with stroke, and sleep instability can promote stroke, thus forming a vicious cycle (Dyken and Im, 2009). Despite the well-known complex relationship between sleep and stroke, scarce data exists about the microarchitecture of REM sleep in relation to stroke onset time.

In this study, we evaluated the microarchitecture of REM sleep in patients with WUS and DTS i.e., REM sleep percentage, AHI during REM sleep, oxygen saturation during REM sleep, and early morning REM duration. Of interest, patients with WUS tended to have a shorter disrupted REM. The disruption was in the form of higher apnea hypopnea index and lower oxygen saturation The AHI during REM sleep was significantly higher in WUS patients than in DTS patients in our study. The mean SO_2_ during REM was significantly lower in WUS patients in comparison to DTS patients. Of note, despite the higher AHI and lower oxygen saturation during REM sleep among those patients, the total arousal index was not significantly elevated. This means that the patients did not get aroused despite the frequent apneas and, therefore, their oxygen saturation was getting worse and that is why they might have developed cerebrovascular ischemia. Thus, the disruption of REM sleep (i.e., in the form of shorter duration, higher AHI and lower SO_2_) seemed to increase the risk for wake-up stroke. To the best of our knowledge, none of the studies in the previous literature compared the microstructure of REM and NREM sleep (i.e., the AHI and SO_2_) in patients with WUS and DTS. Data from previous literature focused on AHI during sleep in general without specification of the stage of sleep during which the apneas or hypopneas occur. Apnea hypopnea index is an indicator of the severity of OSA and measures the number of apneas and hypopneas that occur per hour of sleep (Asghari and Mohammadi, 2013). The previous literature studies reported that the increased AHI increases the risk of stroke, but they did not specify whether the increase in AHI occurred during REM or NREM sleep. For instance, Mohamed et al. studied the difference between the polysomnographic studies between DTS and WUS in 107 patients in Italy, and reported that obstructive sleep apnea was significantly more prevalent among patients with WUS in comparison to patients with DTS (Mohammad et al., 2019). Similarly, Hsieh et al. in their cross-sectional study on 71 patients from Taiwan, reported that OSA was the only risk factor for WUS (Hsieh et al., 2012). Though there was no significant difference in the gender distribution among our patients' groups, there was a meaningful higher number of female patients (i.e., greater than the double) among WUS group. This goes in line with what had been previously reported that women have significantly more REM-related disordered sleep breathing than their male counterparts (Koo et al., 2008). This makes our findings particularly interesting as the group with a greater number of female patients (i.e., WUS group) had significantly higher AHI during REM sleep over the recording period.

Moreover, we found that WUS patients had longer duration of early morning REM than DTS. This finding might suggest that a longer early morning REM might increase the risk to develop WUS. Peter-Derex who stated that the morning peak in the frequency of vascular events might not only be related to circadian variations, but also to REM sleep morning preeminence support this hypothesis (Peter-Derex and Derex, 2019). This is because the last sleep cycles of the night contain more REM sleep, a state of autonomic instability dominated by remarkable fluctuations between parasympathetic and sympathetic influences, whereas NREM sleep is associated with an increase in vagal drive and a decrease in cardiac sympathetic activity (Somers et al., 1993; Baust et al., 1972). REM sleep thus constitutes a period of vascular vulnerability as compared to NREM sleep. This has been highlighted in the setting of myocardial infarction in patients with coronary artery disease (King et al., 1973).

One important point to be noted is that the cross-sectional nature of our study cannot confirm whether the changes we noted are a cause or result of cerebrovascular stroke; particularly that some reports from literature stated that the REM sleep is shortened during the acute phase of stroke (defined as during two to seven days) from stroke onset (Ferre et al., 2013). In our study, the polysomnographic study was performed within the first week of the stroke onset. Thus, the abnormal short REM can be a result of stroke rather than a risk factor.

Another point to be noted is that the long stage 2 sleep noted in our patients indicates that they might have higher rates of cyclic alternating pattern. The cyclic alternating pattern (CAP) is a periodic activity of NREM sleep (especially stage 2), characterized by sequences of transient electro-cortical events that recur at up to 1-min intervals (Parrino et al., 2014). In, CAP represents periods of instability that reflects the brain's effort in preserving and regulating the physiological structure of sleep, and it is associated with frequent transient episodes of hypoxia (Parrino et al., 2014). These transient hypoxic episodes that occur during the CAP cycling were theorized to negatively affect the cerebrovascular musculature and, thus, increase the risk of cerebrovascular stroke (Parrino et al., 2014; Näsi et al., 2012). Therefore, we propose that the long stage 2 sleep might have increased the risk of WUS via increasing the transient hypoxic episodes during CAP.

Regarding PLM, we found that the PLM index and PLM-related arousals were significantly higher among patients with DTS in comparison to WUS patients. To the best of our knowledge, data from previous literature are lacking about the exact relation between PLM and the diurnal pattern of stroke. Our finding might suggest that the PLM index could be a risk factor to develop stroke during morning. This is supported by the data reported by Min Koo et al. who hypothesized that the relation between PLM and stroke is mediated via sympathetic overactivity, oxidative stress, inflammation, hypoxia, and/or metabolic dysregulation which causes daytime hypertension and subsequently might increase the risk of cerebrovascular disease (Walters and Rye, 2009).

## 5 Conclusion

Rapid eye movement (REM) sleep is disrupted in patients with WUS. WUS patients had shorter REM sleep %, longer early morning REM duration, higher AHI index during REM sleep, and lower mean O_2_ saturation during REM in comparison to patients with DTS.

## Author contribution

Prof. Dr.Jaidaa Mekky and Prof.Dr Osama Elkholy conceived of the presented idea. Both. Dr, Jaidaa Mekky and dr.Akram Fawzy developed the theory and performed the study design.The sleep study scoring and revision were performed by Dr. Mekky, Dr. Eman Hamdy and Dr. Akram Fawzy and they took care of the data analysis and profiling of the patients. All authors contributed in writing and revising the manuscript.

## Author contribution

Prof. Dr.Jaidaa Mekky and Prof.Dr Osama Elkholy conceived of the presented idea. Both.

Dr, Jaidaa Mekky and dr.Akram Fawzy developed the theory and performed the study design.The sleep study scoring and revision were performed by Dr. Mekky, Dr. Eman Hamdy and Dr. Akram Fawzy and they took care of the data analysis and profiling of the patients. All authors contributed in writing and revising the manuscript.

## Declaration of competing interest

The authors have declared no conflict of interest.
